# Peer Review of “Evaluating the Financial Factors Influencing Maternal, Newborn, and Child Health in Africa: Tobit Regression and Data Envelopment Analysis”

**DOI:** 10.2196/85383

**Published:** 2025-11-28

**Authors:** Masoud Mahundi

**Affiliations:** 1 University of Dar es Salaam, Dar es Salaam, United Republic of Tanzania

**Keywords:** financial determinants, maternal, newborn, and child health, health care efficiency, Africa, health expenditure, data envelopment analysis, Tobit regression

*This is the peer-review report for “Evaluating the Financial Factors Influencing Maternal, Newborn, and Child Health in Africa: Tobit Regression and Data Envelopment Analysis.”*

## Round 1 Review

### General Comments

Although the title might initially seem misleading, the paper [1] tackles an essential issue of efficiency in delivering maternal, newborn, and child health services in Africa. Given the well-known challenges facing maternal and newborn health in the region, the importance of this study cannot be overstated.

### Specific Comments

#### Major Comments

1. The whole abstract is more about general efficiency in health care systems and less about the financial factors influencing maternal, newborn, and child health (MNCH) in Africa. The title has to reflect what the study actually presents.
2. If the key aim of the study was to determine how financial factors influence MNCH, one would then expect to see, in the abstract, the extent of the influence of financial factors such as health expenditure, coverage index, and expenditure per capita.
3. The Introduction section starts with a presentation of the number of women dying in 2020 and the number of children dying in 2021. These data are not supported with any citations. It is also a little strange that for the number of women dying, the study refers to 2020 data, while for that of children, the study refers to 2021.
4. The Introduction section does not provide sufficient motivation for investigating efficiency in health systems. The first paragraph presents the maternal health challenges, while the second paragraph quickly goes to the methods for establishing efficiency. There is no connection as to why investigating efficiency is necessary.
5. The Introduction section provides a descriptive review of other studies without depth. It lists the different studies without synthesizing them. It would be useful if they were at least lifted up to present issues/themes so it is easy to connect with what the study is about.
6. There is a sentence in the Methods section (Data Sources and Variables) that says “Input, output, and explanatory variables were selected to assess the accuracy of the WHO [World Health Organization]...” Are there three types of variables in your study?
7. What is presented as stages of data envelopment analysis does not go further to describe how the study made use of these stages. Much of the presentations are about what these stages are and sometimes the historical background. It would be useful to put more emphasis on how the study used these stages so it assures the credibility and reliability of the findings.
8. The presented results do not have a clear foundation from the methods. The chain of evidence from the data is lacking, from their processing to their results.
9. The parameters presenting the results are not clearly defined. It says “...26% with a score of 1.” There is no proper introduction of the ranges for a reader to comprehend the meaning of a score of 1. It also states that “...average efficiency score (TE-VRS) across all countries is 0.849 for VRS [variable returns to scale].”
10. The Discussion section needs revising. It does not directly connect to the findings of the study, despite the challenges of the results. The Discussion section further presents a couple of statistics, especially in the first and second paragraphs, without sources or a clear connection to the findings.

## Abbreviations

MNCH: maternal, newborn, and child health
